# 

**Published:** 1919-06

**Authors:** 


### CONTENTS OF THE JUNE ISSUE

Medical Colleges. . . . . . . . . . . . . . . . . . . . . . . . . . . . . . . . . . . . . . . . . 409
Children First. . . . . . . . . . . . . . . . . . . . . . . . . . . . . . . . . . . . . . . . . . . 410
Cost of Sickness. . . . . . . . . . . . . . . . . . . . . . . . . . . . . . . . . . . . . . . . . 412
Spreading Influenza. . . . . . . . . . . . . . . . . . . . . . . . . . . . . . . . . . . . . . 412
Head Injuries . . . . . . . . . . . . . . . . . . . . . . . . . . . . . . . . . . . . . . . . . . 414
A Sanitary Crusade Against Cave Dwellers. . . . . . . . . . . . . . . . 419
Non-Vital Teeth. . . . . . . . . . . . . . . . . . . . . . . . . . . . . . . . . . . . . . . . . 425
Sleeping Sickness. . . . . . . . . . . . . . . . . . . . . . . . . . . . . . . . . . . . . . . 429
Medical Letter-Bag. . . . . . . . . . . . . . . . . . . . . . . . . . . . . . . . . . . . . . 436
Medical Miscellany. . . . . . . . . . . . . . . . . . . . . . . . . . . . . . . . . . . . . 441
Personal. . . . . . . . . . . . . . . . . . . . . . . . . . . . . . . . . . . . . . . . . . . . . . 442
Hospital Notes. . . . . . . . . . . . . . . . . . . . . . . . . . . . . . . . . . . . . . . . . 445
Old Jokes. . . . . . . . . . . . . . . . . . . . . . . . . . . . . . . . . . . . . . . . . . . . . 446

